# Scalable Technology for Adolescents and Youth to Reduce Stress in the Treatment of Common Mental Disorders in Jordan: Protocol for a Randomized Controlled Trial

**DOI:** 10.2196/54585

**Published:** 2024-11-08

**Authors:** Aemal Akhtar, Anne Marijn de Graaff, Rand Habashneh, Dharani Keyan, Adnan Abualhaija, Sarah Fanatseh, Muhannad Faroun, Ibrahim Said Aqel, Latefa Dardas, Chiara Servili, Mark van Ommeren, Richard Bryant, Kenneth Carswell

**Affiliations:** 1 Department of Clinical Neuroscience Division of Insurance Medicine Karolinska Institutet Stockholm Sweden; 2 School of Psychology University of New South Wales Sydney Australia; 3 Department of Mental Health Brain health and Substance Use World Health Organization Geneva Switzerland; 4 Institute for Family Health King Hussein Foundation Amman Jordan; 5 Community Health Nursing Department School of Nursing The University of Jordan Amman Jordan

**Keywords:** mHealth, psychosocial intervention, youths, depression, anxiety, minimally guided digital intervention, low- and middle-income countries, barriers, access, mental health, digital self-help, chatbots, conversational agents, effectiveness, Scalable Technology for Adolescents and Youth to Reduce Stress, randomized controlled trial, Jordan, psychological distress, mental disorders, disability

## Abstract

**Background:**

Young people in low- and middle-income countries encounter significant barriers to accessing mental health support due to various factors, including a substantial treatment gap and limited health care budgets allocated to mental health. Using innovative strategies, such as scalable digital self-help psychological interventions, offers a potential solution for improving access to mental health support. However, digital mental health interventions come with their own set of challenges, including issues related to low user engagement. Chatbots, with their interactive and engaging nature, may present a promising avenue for the delivery of these interventions.

**Objective:**

This study aims to explore the effectiveness of a newly developed World Health Organization (WHO) digital mental health intervention, titled Scalable Technology for Adolescents and Youth to Reduce Stress (STARS).

**Methods:**

A single-blind, 2-arm randomized controlled trial will be conducted nationally across Jordan. Participants will include 344 young adults, aged 18-21 years, currently residing in Jordan. Inclusion criteria are heightened levels of psychological distress as determined through the 10-item Kessler Psychological Distress Scale (≥20). Assessment measures will be conducted at baseline, 1-week post intervention, and 3-month follow-up. Following baseline assessments, eligible participants will be randomized to receive STARS or enhanced usual care. The primary outcomes are the reduction of symptoms of depression and anxiety (Hopkins Symptom Checklist, 25 subscales) at 3-month follow-up. Secondary outcomes include general functioning (WHO Disability Assessment Schedule 2.0), well-being (WHO-5 Well-Being Index), personal problems (Psychological Outcomes Profile), and agency (State Hope Scale subscale).

**Results:**

The study was funded in January 2020 by the Research for Health in Humanitarian Crises Programme (Elhra) and recruitment for the trial started on July 16, 2023. As of November 15, 2023, we randomized 228 participants.

**Conclusions:**

This trial intends to contribute to the growing digital mental health evidence base by exploring technological solutions to address global public health challenges. Given the widespread use of technology globally, even in resource-constrained settings, and the high adoption rates among adolescents and young individuals, digital initiatives such as STARS present promising opportunities for the future of mental health care in low- and middle-income countries.

**Trial Registration:**

ISRCTN Registry ISRCTN10152961; https://www.isrctn.com/ISRCTN10152961

**International Registered Report Identifier (IRRID):**

DERR1-10.2196/54585

## Introduction

Mental disorders are one of the leading causes of disability worldwide, with an estimated 284 million and 264 million people affected by anxiety and depression, respectively [1]. The majority of mental disorders emerge between childhood and young adulthood, before the age of 25 years [2], with a median age of onset at 18 years [3]. The proportion of people with any anxiety disorder and the proportion of those with major depressive disorder that started before the age of 25 years is 7 out of 10 and 4 out of 10, respectively [3].

Although there exists a high prevalence of mental disorders, there remains an equally high treatment gap, with estimates of between 76% to 85% of people—in countries classified by the World Bank’s Development Indicators as “less developed” receiving no treatment [4]*.* This treatment gap is exacerbated by low expenditure on mental health services, with countries allocating on average less than 2% of their health care budgets to mental health*.* Compared to adults, young people are less likely to access mental health services due to reasons such as perceived stigma and limited knowledge about mental health [5]. Additionally, treatment is often designed for adults and may not be appropriate for younger adults [6]. There is a need for innovative approaches to increase access to mental health care. This need may be more pressing for conflict-affected populations, where the World Health Organization (WHO) estimates suggest around 1 in 5 people affected by conflict in the previous 10 years currently experience a mental disorder [7].

Research on the delivery of psychological interventions by nonspecialists, using task shifting, shows promising results [8]. Yet, while the evidence for the effectiveness of these interventions for youths is growing, difficulties remain in accessing evidence-based interventions, particularly in low- and middle-income countries (LMICs) [9,10]. This highlights the need for evidence-based psychological interventions that can be used in settings that are resource-pressured [11]. Such interventions should be suitable for scale-up, as well as being appropriate for diverse sociocultural groups. A number of these interventions have been developed for adult populations, with meta-analytical evidence suggesting their effectiveness [12]. However, there are fewer interventions that have been developed and trialed for younger populations with the resulting evidence being less concrete [9]. Digital solutions may be one way to mitigate some of the access and resource challenges for young people [8].

Digital mental health (also called e-mental health) interventions refer in part to the use of electronic devices such as smartphones to support the provision of mental health care. In high-income settings, some guided self-help interventions have been found to be as effective as face-to-face interventions in adults [13,14]. Additionally, these interventions have been shown to reduce symptoms of mental disorders in routine care [15]. Similar delivery models have also been shown to be effective in more resource-constrained, middle-income countries and with conflict-affected populations [16-18]. This compelling evidence has led to the inclusion of digital mental health interventions in a number of countries’ national mental health strategies and treatment guidelines [19]. In addition, WHO guidelines on mental health promotive and preventive interventions for adolescents and youths recommend the use of digital mental health interventions [20].

While the existing evidence for digital mental health interventions for youths suggests such interventions may be beneficial [21-23], the public availability of evidence-based interventions remains limited [9,24,25]. Additionally, while research on the efficacy of internet-delivered interventions is developing [21,26,27], studies beyond high-income English-speaking countries remain sparse, with the exception of China, where some studies have been completed [22,23]. However, digital mental health interventions may be a useful approach for increasing access to treatment because the use of electronic devices, internet, and social media continues to rise in LMICs, especially among youths [28-30]. This suggests the importance of developing, adapting, and determining the effectiveness of these types of interventions for younger populations, particularly in low- and middle-income and conflict-affected settings.

Implementation of digital mental health approaches is not without its challenges, as they introduce particular complexities associated with the use of digital platforms, such as the complexity and cost of building and maintaining some smartphone apps, which may lead to barriers in scale-up attempts [31]*.* A potential way to address these barriers is to explore the benefits of different delivery systems for online interventions. One such delivery method is using conversational agents, more commonly known as chatbots. Chatbots may provide a potential alternative and highly engaging method to deliver such interventions. Chatbots are text-based automated conversational agents that give the impression of speaking to a human, although the user is aware beforehand, that they are speaking with a computer program. They may use machine learning and respond to user inputs or use a decision tree format with users following a defined path. Preliminary studies exploring the effectiveness of chatbots on mental health outcomes are promising [32,33] and suggest they may be a practical way of providing mental health care as they can (1) use existing and widely available software infrastructures, and (2) may increase engagement due to the conversational nature of delivery.

For these reasons, the WHO has designed and developed a chatbot based on decision tree logic for youths, entitled Scalable Technology for Adolescents and Youth to Reduce Stress (STARS) [34]. This protocol outlines the first randomized controlled trial (RCT) aiming to test the effectiveness of STARS, a guided self-help mental health chatbot intervention among youths residing in Jordan, including Syrian refugees. This study builds and adds to the current literature as it aims to test a chatbot as an innovative delivery approach that may overcome some of the barriers and challenges involved in digital mental health interventions, within a middle-income, Arabic-speaking country, where specialist care for mental health is particularly limited and there is a high proportion of refugees per capita. Testing in this setting will demonstrate whether such an intervention aimed at youths may be a feasible model for scaling up much-needed services. This trial follows preliminary work on the development of the STARS intervention, including the cultural adaptation of STARS to the Jordanian context (not yet published), and a completed pilot trial to determine cultural acceptance and appropriate research methods. The primary aim of this study is to determine the effectiveness of the STARS intervention in alleviating symptoms of depression and anxiety among individuals aged 18-21 years residing in Jordan, compared to enhanced usual care (EUC). Secondary aims include assessing the effectiveness of STARS in improving levels of psychological distress, self-defined problems, general functioning, agency, and subjective well-being.

## Methods

### Design

We will conduct a 2-arm, single-blind, superiority RCT comparing the STARS intervention to EUC in 344 study participants. Multimedia Appendix 1 presents the Standard Protocol Items: Recommendations for Intervention Trials (SPIRIT) checklist and Multimedia Appendix 2 presents the trial registration data.

### Setting

The project will take place at a national level in Jordan. Jordan is a middle-income country with limited resources allocated to mental health care. Specialist care for mental health problems is especially limited, with 1.04 psychiatrists, 2.97 mental health nurses, 0.11 psychologists, and 0.01 occupational therapists available per 100,000 people [35]. In addition, Jordan hosts the second most refugees per capita, globally, with the majority originating from Syria [36], further straining available mental health care. The majority of people residing in Jordan have access to the internet, with the World Bank reporting 83% of the population able to access the internet in 2021 [37] ensuring that a digital mental health intervention would be both accessible and scalable. The study will be implemented by the Institute for Family Health (IFH), a nongovernmental organization operating across Jordan.

### Participants and Recruitment

Participants will include youths aged 18 to 21 years residing in Jordan. Recruitment will be conducted through various means, including online advertisements and social media channels, adverts, and posters hosted in community centers, primary health care facilities, universities, and other areas frequented by this age group. During the recruitment process, potential participants will be able to access the study through either a QR code or a web link. Inclusion criteria for potential participants are (1) youths aged 18 to 21 years; (2) living in Jordan; (3) scores 20 or higher on the Kessler Psychological Distress Scale, 10-item version (K10) [38], indicative of moderate levels of psychological distress; and (4) having access to a device for intervention delivery or willingness to use one at a participating center. Exclusion criteria are (1) persons not between the ages of 18 and 21 years, and (2) imminent risk of suicide.

### Ethical Considerations

The RCT has been approved locally by the research committee of the School of Nursing, University of Jordan (PF.22.9 on March 23, 2022) and by the WHO ethics review committee (ERC; ERC.0003729 on July 1, 2022). Informed consent for participation in the trial will be taken prior to screening. Upon initially accessing the STARS website through the recruitment material, participants will receive information regarding the purpose of the study, an explanation of the assessments and study procedures, and information on the 2 interventions being trialed; STARS and EUC. This written information is additionally provided in a short, 3.5-minute animated video. They will subsequently be invited to complete the screening assessment. Participants will be remunerated JOD 4 (US $5.64) upon completion of the postassessment and again following the 3-month follow-up assessment.

### Study Procedures

**Figure 1:**
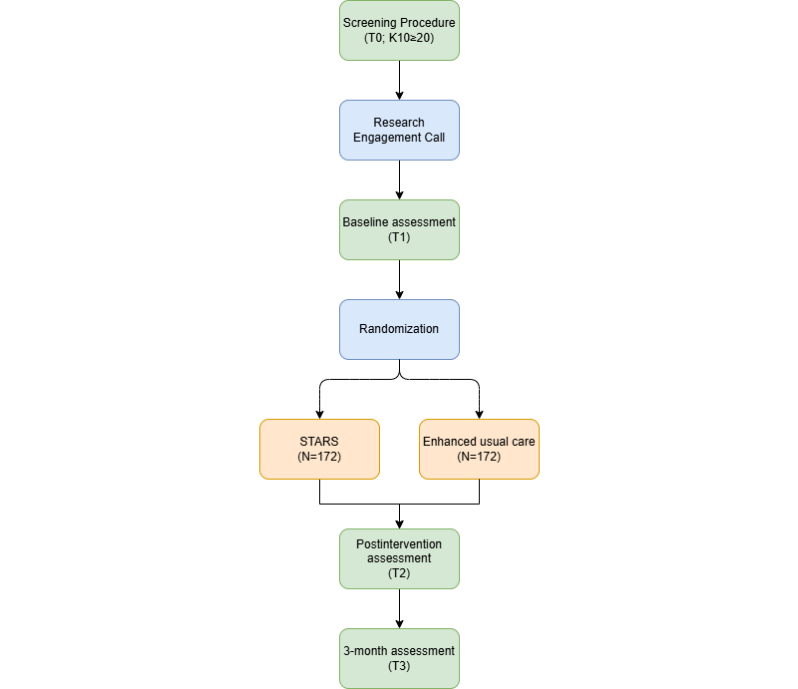
Study flowchart. K10: Kessler Psychological Distress Scale, 10-item version; STARS: Scalable Technology for Adolescents and Youth to Reduce Stress.

Figure 1 presents a study flowchart outlining the study procedure. Prior to the commencement of the trial, STARS underwent a rigorous cultural adaption to ensure that the translation and material contained in STARS were relevant to the Jordanian context. This culturally adapted version of STARS was subsequently piloted in a feasibility RCT to ensure the safety, acceptability, and feasibility of the proposed research methodology (not yet published).

Participants will be directed to take part in the screening procedure after accessing the online software tool, Qualtrics, through the research project’s website and reading through the study information outlined earlier. As part of the screening procedure, participants will first be asked their country of residence and age, administered the K10, and administered the screening questions for imminent risk of suicide. Participants outside of Jordan, not between the ages 18-21 years, or screening negative on the K10 will be informed that they are not eligible for participation and will be invited to contact the implementing team if they have any questions or would like to receive information on psychosocial services available close to them. If participants are excluded due to screening positive for imminent risk of suicide, a message will appear that they may need additional mental health support along with signposting to a nationally recognized helpline.

If participants meet all inclusion criteria, they will be invited to provide their contact information (phone number and/or email address). Immediately after having completed the screening or on the next working day, participants will be contacted by a member of the research team to answer any questions regarding the study. Participants will then receive a personalized link to the baseline questionnaires.

All subsequent assessments will also be completed remotely by participants either on their own private devices, or devices that will be made available in private rooms at the IFH health care centers. The baseline assessment will include the following instruments: Hopkins Symptoms Checklist, 25 (HSCL-25) [39], WHO Disability Assessment Schedule 2.0 (WHODAS 2.0) [40], WHO-5 Well-Being Index (WHO-5) [41], Psychological Outcomes Profile (PSYCHLOPS) [42], and the agency subscale of the State Hope Scale (SHS-A) [43]. In addition, participants will be asked about their previous mental health care use both related to services and prescribed medicines. Following the baseline assessment, participants will be randomized to either receive STARS or EUC.

The postintervention assessment is scheduled to take place at 8 weeks following baseline assessment completion for all participants. The postintervention assessment will include the HSCL-25, K-10, WHODAS 2.0, WHO-5, PSYCHLOPS, SHS-A, the Client Satisfaction Questionnaire adapted to internet-based interventions [44], and questions about whether they accessed mental health care during the trial. The final assessment will be conducted in 3 months following the postassessment, or 20 weeks after the baseline assessment, and will include the HSCL-25, K-10, WHODAS 2.0, WHO-5, PSYCHLOPS, SHS-A, and questions pertaining to any mental health services received between the postintervention assessment and the time of the 3-month assessment. Participants will receive up to 3 reminder messages and/or phone calls spread out over 2 weeks including their personalized link to the assessment. Participants will be remunerated JOD 4 (US $5.64) upon completion of the postassessment and again following the 3-month follow-up assessment. Table 1 presents a SPIRIT diagram outlining the assessment and intervention time points of the trial.

**Table 1:** SPIRIT^a^: Schedule of enrollment, interventions, and assessments for the STARS^b^ trial.

		Measures	Enrollment (–T1)	Screening (T0)	Baseline (T1)	Intervention	Postassessment (T2; 8 weeks)	3-month follow-up (T3; 20 weeks)
**Enrollment**								
	Informed consent		✓					
	Eligibility screen			✓				
	Allocation				✓			
**Interventions**								
	STARS				✓	✓	✓	
	ETAU^c^				✓	✓	✓	
**Assessments**								
	Psychological distress	K10^d^		✓			✓	✓
	Depression symptoms	HSCL-25^e^			✓		✓	✓
	Depression symptoms	PHQ-2^f^				✓		
	Anxiety symptoms	HSCL-25			✓		✓	✓
	Functioning	WHODAS 2.0^g^			✓		✓	✓
	Subjective well-being	WHO-5^h^			✓		✓	✓
	Perceived problems	PSYCHLOPS^i^			✓		✓	✓
	Agency	SHS-A^j^			✓		✓	✓
	User satisfaction	CSQ-I^k^					✓	
	Mental health care use	2 items			✓		✓	✓

^a^SPIRIT: Standard Protocol Items: Recommendations for Intervention Trials.

^b^STARS: Scalable Technology for Adolescents and Youth to Reduce Stress.

^c^ETAU: enhanced treatment as usual.

^d^K10: Kessler Psychological Distress Scale, 10-item version.

^e^HSCL-25: Hopkins Symptoms Checklist, 25 subscales.

^f^PHQ-2: Patient Health Questionnaire, 2-item version.

^g^WHODAS 2.0: WHO Disability Assessment Schedule 2.0.

^h^WHO-5: WHO-5 Well-Being Index.

^i^PSYCHLOPS: Psychological Outcomes Profile.

^j^SHS-A: State Hope Scale, agency subscale.

^k^CSQ-I: Client Satisfaction Questionnaire adapted to internet-based interventions.

### Randomization and Blinding

Participants will be randomized to receive either STARS or EUC at a 1:1 allocation ratio following the baseline assessment. The randomization sequence will be developed through Qualtrics software and will be automated and embedded in the baseline questionnaire, with participants having their group allocation conveyed to them immediately upon completion of the assessment. Given the high proportion of Syrian refugees and other populations residing in Jordan, randomization will be stratified on a 1:1 ratio (Jordanian or Palestinian vs Syrian or other nationalities). Given that questionnaires are self-administered online, the outcome assessments will not be blinded.

### Interventions

#### Scalable Technology for Adolescents and Youth to Reduce Stress

The STARS intervention has been described in detail elsewhere [34]. The intervention is guided, brief, and consists of 10 chatbot sessions that have been developed from evidence-based techniques to address emotional distress experienced by youths. Participants are encouraged to complete 2 chatbot sessions a week spread out over 5 weeks, with each session taking approximately 20-25 minutes to complete. Upon completion of a chatbot session, the next chatbot session is unlocked immediately. During initial registration, participants will be able to opt-in to receive push notifications to receive reminders to complete prior chatbot sessions, to be alerted when a new session is available, and yet to be started, or when they have not accessed the intervention for some time. There are several notifications to improve engagement of participants with the chatbot. These notifications are described in Multimedia Appendix 3. The STARS intervention is being delivered using the OpenDialog automated conversation system.

Prior to use in this study, a cultural adaptation of the intervention took place to ensure that the chatbot was relevant to the Jordanian context (not yet published). As part of the cultural adaptation procedures, a prior iteration of the chatbot was translated from English to Arabic. This was then tested with youths using commonly used adaptation procedures outlined by WHO and detailed in a recently released manual [45]. These include techniques such as “cognitive interviewing,” a process that explores how relevant, understandable and acceptable an intervention is. Additionally, focus groups were used to understand the context and how to maximize the reach of STARS in Jordan. After the development of an updated STARS, there was an additional round of user testing to further refine the included content, with a focus on the wording and presentation of the content. The adapted version, called SALAM, will be used in this trial.

STARS was based on a cognitive behavioral therapy framework to deliver psychological content aimed at addressing the broad mental health needs reported by adolescents and youths. Cognitive behavioral therapy is recommended as an evidence-based mental health treatment for youths with emotional disorders according to WHO guidelines [20]. Lesson 1 (“Let’s get started”) provides participants with an overview of the intervention and an explanation of privacy and confidentiality. Lesson 2 (“Emotions are not our enemies”) provides participants with psychoeducation about emotions. Lessons 3 and 4 (“Breathe to relax”—parts 1 and 2) teach participants emotion regulation techniques, such as slow breathing. Lessons 5 and 6 (“What we do can change how we feel”—parts 1 and 2) focuses on behavioral activation. Session 7 (“Managing problems”) focuses on problem management techniques. Lessons 8 and 9 (“Talking kindly to yourself”—parts 1 and 2) teach participants about thought challenging. Finally, lesson 10 (“Put your skills together”) is focused on consolidating learnings and relapse prevention [34]. The techniques are taught by SALAM that sends brief text messages to which participants can respond using predefined response options consisting of prewritten text and emojis. The use of the response buttons is “practiced” during the first lesson. In some lessons, there is an opportunity to give a free-text response, for example, when participants are asked to specify a goal for the intervention that has not been predefined by the chatbot. Additionally, useful tools such as audio and video demonstrations (eg, breathing techniques, problem management) and practice quizzes will be available for use anytime, as will the ability to go back to a previous session. This can be done by navigating to the “session menu” where participants can select the lesson they would like to repeat. Throughout the intervention, users will be encouraged to practice and apply these skills in their day-to-day lives.

The training and materials for the guided component of the chatbot intervention are based on a guided self-help approach developed by WHO and used in several studies [17,18,46]. It consists of 5 weekly telephone calls by trained, nonspecialist e-helpers. The support calls last approximately 15 minutes each to provide participants with support and guidance to implement the STARS intervention techniques and to use the STARS intervention chatbot. E-helper support is strongly protocolized and outlined session-by-session for each support phone call in an e-helper manual. E-helpers are trained on the STARS intervention and research protocols during a 5-day training delivered by IFH. The training includes knowledge about psychological distress and other mental health problems, communication skills, providing support, responding to distress, and procedures for adverse events (AEs) including referral procedures. E-helpers can monitor their participants’ progress through the OpenDialog system and can view some of the participants’ responses. These are limited to specific parts that are relevant for the e-helper, such as responses to the mood check, the goal a participant selected, and free-text fields. Free-text responses will be monitored on a daily basis for any indications that the participant is unsafe (eg, in case a participant expresses suicidal ideation in a free-text field or suggests a harmful goal to work on in the intervention). E-helpers will be supervised on a weekly basis by a clinical supervisor based at IFH, with additional support to the project team from WHO. Clinical group supervision will ensure fidelity of the e-helper support calls, discussion of challenging situations when providing telephone support, and e-helper self-care. The clinical supervisor will also listen to 5% of e-helper support calls to provide feedback on the delivery of the support calls.

To evaluate treatment fidelity, a random sample (5%) of e-helper support calls will be audio recorded. Participants will be asked for verbal consent prior to recording. Recordings will be evaluated according to a treatment fidelity checklist. This information has also been added to the consent form.

#### Enhanced Usual Care

The EUC intervention will include basic psychoeducation regarding depression and anxiety, as well as resources on available evidence-based care available in Jordan. This will be delivered through a website where participants register to gain access. The information provided to those in EUC will also be provided during the psychoeducation session to those enrolled in the STARS intervention. Available resources will include a list of organizations that offer evidence-based mental health and psychosocial support in Jordan—as this study is taking place at a national level, a list has been created of the resources available across the country so that those in need can access services close to their physical location.

### Measures

All assessments will take place remotely and be hosted through Qualtrics, a secure data collection online platform.

#### Screening Measures

The screening assessment consists of the K10 to assess levels of psychological distress [38] and screening questions to determine the imminent risk of suicide informed by the WHO mhGAP Intervention Guide 2.0 [47]. The K10 assesses general psychological distress and consists of 10 items corresponding to symptoms of depression and anxiety experienced in the preceding 30 days. Items are rated on a 5-point Likert scale ranging from 1=none of the time to 5=all of the time, with scores determined through the sum of the items (range 10-50). Higher scores are indicative of higher levels of distress; a cutoff score of 20 will be used to determine inclusion as it has been previously shown to indicate moderate to significant levels of distress [48]. The K10 will also be used as a secondary outcome measure. The imminent risk of suicide, as defined by the WHO Mental Health GAP program, will be screened through 3 questions [47]. Participants will be asked to indicate whether they have had thoughts of suicide in the preceding 30 days and if a positive response is recorded, follow-up questions regarding prior attempts or future plans will be asked. Participants who indicate imminent risk as determined by the follow-up questions will be excluded from the study and provided with on-screen information directing them to support services.

#### Primary Outcome

The primary outcome will be levels of depression and anxiety as determined through the HSCL-25 [39]. The HSCL-25 is a 25-item questionnaire consisting of 2 subscales assessing symptoms of depression and anxiety; 15 items correspond to symptoms of depression and 10 correspond to symptoms of anxiety. The validity and psychometric properties of the questionnaire have been well established in both adults and youths or adolescents [49,50]. Additionally, an Arabic version of the HSCL-25 has been previously validated [51] and has been used successfully among adults in Jordan during a previous study determining the effectiveness of a scalable psychological intervention [52].

#### Secondary Outcomes

The WHODAS 2.0 will be used to assess levels of impaired functioning [40]. The measure assesses general disability people experience in their daily lives over the prior 30 days across 6 domains: self-care, getting along with others, cognition, mobility, participation, and life activities. The 12-item version will be used, which includes 2 contributing items from each domain. Difficulties are scored on a 5-point scale ranging from 0=none to 4=extreme, with total scores ranging from 0 to 48. The measure has been widely validated globally, with specific psychometric studies taking place in youth populations [53] and on the Arabic version [40,54]*.*

The PSYCHLOPS measure will be used to understand participants’ primary problems and how they impact daily functioning, and to track improvements of these problems over the course of the study [42]. The measure includes 4 items spanning 3 domains: problems, functioning, and well-being. Free-text responses are provided for the problem and functioning domains, with impacts on daily life being scored on a 6-point ordinal scale. Posttreatment and follow-up versions of the measure include an overall valuation question to determine the improvement of these problems. The PSYCHLOPS has been validated in a number of contexts [55,56].

Psychological well-being and quality of life will be assessed through the WHO-5 [41]. The WHO-5 measures subjective positive well-being across 5 items scored on a 6-point Likert scale, with responses ranging from 0=at no time to 5=all of the time. Total scores range from 0 to 25, with higher scores indicating higher levels of well-being. The WHO-5 has been used globally in research and across a number of clinical settings [41] and has previously been validated in an Arabic sample [57].

The SHS-A will be used to assess perceived agency [43]. The agency subscale consists of 3 items observing perceived levels of goal-directed energy measured on an 8-point Likert scale ranging from 1=definitely false to 8=definitely true. The potential mediation effect of agency on treatment outcomes will be explored in the secondary analysis.

#### Other Measures

To capture any mental health services that participants received prior to or during the study period, 2 items will be included in postassessments to capture information on the use of psychological or counseling services and medications (each item answered with yes or no).

Those randomized into the STARS intervention will complete the Patient Health Questionnaire, 2-item version (PHQ-2) [58] during every other session. The PHQ-2 has been shown to allow for the detection and monitoring of depression over time and will allow the research team to identify potential worsening of symptoms in participants during the intervention phase [58]. The measure inquiries about symptoms of depression over the past 2 weeks, with items scored on a 4-point Likert scale ranging from 0=not at all to 3=nearly every day, with scores ranging from 0 to 6.

Finally, user satisfaction of the STARS intervention will be assessed through a version of the Client Satisfaction Questionnaire, a scale intended to measure client satisfaction with health services, adapted specifically for web-based health interventions [44]. The questionnaire includes 8 items scored on a 4-point Likert scale ranging from 1=does not apply to me to 4=does totally apply to me. The assessment will be offered to participants during the post-intervention assessment as an optional additional questionnaire.

### Sample Size

A total of 344 participants will be included in the RCT. This sample size calculation was based on the results of prior trials exploring the effectiveness of comparable digital interventions which report effect sizes (Cohen *d*) ranging from 0.48 to 0.56 [17,18,59]. In addition, a similar effect size was used to determine the sample size for a similar RCT observing the effectiveness of a WHO-developed digital health intervention, based on a conservative, yet clinically significant effect size of 0.5 [60]. Assuming an effect size of 0.5 at a 3-month follow-up (power=90%; α=0.05, 2-sided), the trial would require 172 participants (STARS=86 and EUC=86). Given the high attrition rates reported in digital intervention studies, a conservative attrition rate of 50% was assumed. This attrition rate was informed by the results of the feasibility RCT and comparisons with protocols for similarly designed studies.

### Statistical Analysis

To measure baseline differences between the 2 treatment arms, 2-tailed *t* tests will be conducted for continuous variables and chi-square tests for categorical data. To determine the effectiveness of the STARS intervention, intention-to-treat analysis will be conducted to compare the mean difference between STARS and EUC at baseline, post intervention, and 3-month follow-up. Differential change over time between treatment arms will be assessed using linear mixed models that accommodate missing data at subsequent assessments and provide differential slopes of trajectories between conditions. Hierarchical linear models study treatment effects because this allows the number of observations to vary between participants and handles missing data by using maximum likelihood estimation methods. Missing data will be assumed to be random if participants retained and not retained at 3 months do not differ on baseline characteristics. Fixed (intervention and time of assessment) effects and their interactions will be entered in unstructured models to yield indices of the relative effects of the treatments; time of assessment included baseline, post treatment, and 3-month follow-up. Analyses will focus on the primary outcome and secondary measures, with the main outcome time point being the 3-month follow-up relative to baseline. A subsequent per protocol completers analysis will be conducted, only including those who completed the 3-month follow-up, in order to assess the robustness of the primary analysis.

In addition to these analyses, the role of potential moderators and mediators, such as agency, on outcomes will be explored independently using the PROCESS macro. Two-tailed tests with a significance level of *P*<.05 will be reported for all analyses.

### Data Management, Trial Monitoring, and AE Reporting

All assessment data will be collected electronically in Qualtrics. Participants will receive personalized Qualtrics links for their baseline, post-, and 3-month follow-up assessments so that assessments can be linked to each other. Only the research team will have access to the data in Qualtrics. Deidentified data stored from the STARS intervention (eg, PHQ-2 scores, free-text responses, session completion date) are available to e-helpers and the research team through the OpenDialog website. Data collected online will be held securely and separately from study data, according to the relevant data protection laws. Data will be deidentified and stored confidentially at the University of New South Wales after transferring to an SPSS database.

An identifying key list (a list connecting names to numbers) will be kept by the project manager at IFH. All downloaded electronic data will be pseudonymized and stored on password-protected computers. Hardcopy files of the study, such as supervision notes, will be stored in locked cabinets at IFH. No identifiable data will be used in publications or presentations. Trial monitoring will be done by WHO staff on a biweekly basis to ensure the accuracy of data storage and output.

AEs or serious AEs (SAEs) refer to any undesirable experience of a participant during the trial, whether or not considered related to the trial procedures or to the STARS or EUC interventions. All AEs or SAEs reported spontaneously by participants or observed by the research team or e-helpers will be reported to the IFH clinical supervisor for immediate action and resolution where required. All SAEs will be reported to the local ERC within 24 hours of being informed about the event, and a summary of AEs will be reported to the local ERC every three months. The local ERC will review any SAEs as soon as possible and the summary of AEs during their regular meetings. They will determine any appropriate action with respect to ongoing study conduct. Additionally, all SAEs will be reported to the WHO ERC. As STARS is nonpharmacological and there exists a broad evidence base for the safe use of e-interventions and therefore a low risk for adverse effects, no Data Monitoring Safety Board will be installed for this study. Adverse events will be monitored and reported to the local ethics committee.

Any modifications to the protocol that may impact the conduct of the study, the potential benefit of the participant, or may affect participant safety will require a formal amendment to the protocol. Such amendment will be reviewed and approved by the local ethics committee of the University of Jordan and by the WHO ERC prior to implementation. Administrative changes are minor corrections and/or clarifications that have no effect on the way the study is to be conducted. These administrative changes will be documented in a memorandum and communicated to the WHO ERC.

### Safety Considerations

The therapeutic techniques included across the 10 lessons are evidence-based, and therefore, it is unlikely that participants in the intervention will experience an increase in psychological distress. The PHQ-2 will be administered in the STARS intervention once a week (every other lesson) and will be monitored by e-helpers to track participants’ levels of distress during the intervention phase of the trial.

The development of the STARS intervention was intended to minimize the risk of potential adverse events. Of particular focus was to minimize the loss of privacy or confidentiality of intervention participants. Preliminary work revealed that participants may use shared devices to access the intervention, which led to STARS being developed on a website as opposed to an app, that would require a log-in each time the intervention is accessed. Additionally, during the guided components of the interventions, participants are asked to indicate a codeword during their onboarding that could be used by the e-helpers to ensure that the person on the other end of the line is those accessing STARS. Furthermore, the chatbot is transparent throughout each lesson that they are a program and not a person. Lastly, e-helpers check digital reports on session use, which include responses to open text fields; responses are analyzed to identify responses that may indicate concern for participants’ well-being and allow for the AE or SAE procedures to be followed. The e-helpers and research staff will be trained in the AE or SAE procedures and subsequent referral pathways prior to the start of the trial.

### Scalability of STARS

STARS was developed with potential scalability in mind for future implementation initiatives if the intervention is found to be effective. The chatbot uses a decision tree format which leads users on a defined path through the lessons. The choice to use a decision tree chatbot was in part informed by the technical feasibility of scaling up the intervention across a range of contexts in the future, as it does not require the development or maintenance of a bespoke website or app and can be used on many existing widely available platforms. WHO aims to release the STARS decision tree script and content as open access on the basis of pooled positive results from 2 trials, meaning that STARS could be adapted and implemented on different software platforms and in different contexts. Furthermore, having defined lessons, and a decision tree format will allow for easier reproducibility. Together these design features may be more cost-efficient and support more rapid uptake and sustained use of the intervention over time, compared to a bespoke website or app which may require additional maintenance and support.

## Results

The study was funded by the Research for Health in Humanitarian Crises Programme (47467) in January 2020. Recruitment for the trial commenced on July 16, 2023, and as of November 15, 2023, a total of 228 participants have been randomized. The expected end date of data collection is July 2024. To date, no data analysis has been conducted.

## Discussion

### Brief Summary

Given the immense mental health treatment gap that exists globally, which is more pronounced in LMICs, scalable psychological interventions that can alleviate psychological distress and symptoms of mental health conditions are needed. This is particularly critical for youths, as they often face reduced access to mental health services due to the stigma associated with seeking help and the fact that many existing services are primarily designed for adults.

Digital health solutions can be particularly useful in bridging this gap. This study intends to contribute to the ongoing efforts of developing and evaluating potentially effective digital mental health solutions to minimize the mental health treatment gap by exploring the effectiveness of a novel digital mental health intervention in the form of a chatbot. The goal of this trial is to conduct a single-blind, 2-armed RCT among 344 young adults aged 18-21 years with heightened psychological distress in Jordan to evaluate the effectiveness of a newly developed WHO digital mental health intervention (STARS) in reducing symptoms of depression and anxiety versus EUC. STARS uses an engaging format to provide education and techniques to help reduce psychological distress, such as symptoms of depression and anxiety experienced by youths and adolescents. Recruitment for the trial began on July 16, 2023. Should the intervention demonstrate a decrease in symptoms of depression and anxiety at 3-month follow-up, it would support the potential for implementation of this new scalable psychological intervention for young people. Results from this trial are expected to expand the current range of available scalable psychological interventions.

### Limitations

Limitations to this study will be the potentially high attrition rates that are a common challenge in eMental health research [32] and the lack of long-term follow-up assessments (ie, beyond 3-month follow-up) to evaluate long-term outcomes of the intervention. Both interventions (STARS and EUC) require participants to have access to a device for intervention delivery (or to be willing to use one at a participating center), which may limit the generalizability of findings.

### Conclusions

If the intervention is found to be effective through this trial and an upcoming secondary trial, the WHO aims to release its contents through open-access channels. This will help toward realizing the scalability of the intervention and will provide implementers with access to evidence-based content that can be further adapted and tested in novel contexts. Furthermore, by providing evidence of the effectiveness of this chatbot-based intervention, this study can pave the way for a more diverse range of options for those seeking mental health support. Collectively, this study aims to further develop the evidence base concerning the effectiveness of digital and self-help tools among adolescents and youths. If effective, it is expected to potentially have a substantial impact on shaping future policy decisions regarding the availability of resources for mental health care, ultimately leading to more inclusive and efficient mental health services for those in need.

## Appendix

Multimedia Appendix 1 The SPIRIT (Standard Protocol Items: Recommendations for Interventional Trials) Checklist.

Multimedia Appendix 2 The World Health Organization Trial Registration Data Set.

Multimedia Appendix 3 Overview of notifications used to improve user engagement.

## Abbreviations

AE: adverse event
ERC: ethics review committee
EUC: enhanced usual care
HSCL-25: Hopkins Symptoms Checklist, 25 subscales
IFH: Institute for Family Health
K10: Kessler Psychological Distress Scale, 10-item version
LMIC: low- and middle-income country
PHQ-2: Patient Health Questionnaire, 2-item version
PSYCHLOPS: Psychological Outcomes Profile
RCT: randomized controlled trial
SAE: serious adverse event
SHS-A: State Hope Scale, agency subscale
SPIRIT: Standard Protocol Items: Recommendations for Intervention Trials
STARS: Scalable Technology for Adolescents and Youth to Reduce Stress
WHO: World Health Organization
WHO-5: WHO-5 Well-Being Index
WHODAS 2.0: WHO Disability Assessment Schedule 2.0

## Data Availability

Data generated during this study will be available following the completion and publication of the trial from RB (r.bryant@unsw.edu.au) upon reasonable request.
