# Supplementary material for: Scalable Technology for Adolescents and Youth to Reduce Stress in the Treatment of Common Mental Disorders in Jordan: Protocol for a Randomized Controlled Trial
Source: JMIR Res Protoc. 2024 Nov 8;13:e54585. doi: 10.2196/54585 (PMC11584552; doi:10.2196/54585)
Supplement: Multimedia Appendix 2 [file resprot_v13i1e54585_app2.docx]

| *Trial registration data* |  |
| --- | --- |
| Data category | Information |
| Primary registry and trial identifying number | ISRCTN; ISRCTN10152961 |
| Date of registration in primary registry | 15^th^ of May 2023 |
| Secondary identifying numbers |  |
| Source(s) of monetary or material support | Enhancing Learning & Research for Humanitarian Assistance, ELRHA |
| Primary sponsor | World Health Organization |
| Secondary sponsor(s) |  |
| Contact for public queries | Dr. Kenneth Carswell, [carswellk@who.int](mailto:carswellk@who.int) |
| Contact for scientific queries | Professor Richard Bryant, School of Psychology, University of New South Wales, Sydney, Australia |
| Public title | Testing an e-mental health intervention for youth in Jordan |
| Scientific title | Scalable technology for adolescents and youth to reduce stress (STARS) - Randomised controlled trial |
| Countries of recruitment | Jordan |
| Health condition(s) or problem(s) studied | Common mental disorders; Depression; Anxiety |
| Intervention(s) | Intervention: STARS |
|  | Control: enhanced care as usual |
| Key inclusion and exclusion criteria | Ages eligible for study: 18-21 years years Sexes eligible for study: both |
|  | Inclusion criteria:  1. Any person living in Jordan who can read English or Arabic  2. Is between the ages of 18 and 21 years  3. Is experiencing moderate levels of psychological distress as determined by a score of 20 or above on the Kessler distress scale, 10-item version (K10)  4. Has access to a device for intervention delivery or is willing to use one at a participating centre |
|  | Exclusion criteria:  1. Persons under 18 years of age or over 21 years of age  2. People at imminent risk of suicide (as defined by the mhGAP intervention guide) |
| Study type | Primary study design: Interventional |
|  | Allocation: randomized intervention model. |
|  | Study type: Treatment |
| Date of first enrolment | 06/07/2023 |
| Target sample size | 344 |
| Recruitment status | Completed |
| Primary outcome(s) | 1. Symptoms of anxiety (Hopkins Symptom Checklist anxiety subscale; HSCL-25)  2. Symptoms of depression (HSCL-25 depression subscale) |
| Key secondary outcomes | 1. Psychological distress (Kessler Psychological Distress scale; K10)  2. Symptoms of depression (Patient Health Questionnaire; PHQ-2) [only during STARS intervention]  3. Functioning (WHO Disability Assessment Schedule; WHODAS 2.0)  4. Subjective wellbeing (WHO Well-Being Index; WHO-5)  5. Self-identified problems (Psychological Outcomes Profiles; PSYCHLOPS)  6. Agency (agency subscale of the State Hope Scale; SHS-A)  7. Mental health care use (2 items)  8. User-satisfaction questionnaire (Client Satisfaction Questionnaire; CSQ-I) [only at post-assessment] |
