# Supplementary material for: Scalable Technology for Adolescents and Youth to Reduce Stress in the Treatment of Common Mental Disorders in Jordan: Protocol for a Randomized Controlled Trial
Source: JMIR Res Protoc. 2024 Nov 8;13:e54585. doi: 10.2196/54585 (PMC11584552; doi:10.2196/54585)
Supplement: Multimedia Appendix 3 [file resprot_v13i1e54585_app3.docx]

| Notification name | Trigger | Rule | English copy |
| --- | --- | --- | --- |
| Onboarding 1 | Participant hasn't created an account in 48hrs | Send a reminder | "Hi {{first name}}, Salam here! Please follow this link to create your account and start chatting with me! [link]" |
| Onboarding 2 | Account not created withing 96hrs | Send a reminder | "Hi {{first name}}, Salam here! I noticed that you did not yet create your account. Follow this link to sign up and start chatting with me! [link]" |
| Onboarding 3 | Account not created in 7 days | Send a reminder | "Hi {{first name}}, it's Salam again! Would you be interested in learning some cool new self-help skills? Follow this link to create your account: [link]" |
| Onboarding 4 | Account not created in 30 days | Send a reminder | "Hi {{first name}}, this is Salam! Please know that you can still create an account! Follow this link and start chatting with me right away: [link]" |
| Nudge to complete session | Participant has not completed session within 48hrs | Send a reminder to continue session | "Hi {{first name}}, Salam here! I noticed that you didn't complete your last session. Head back to the app to finish it, [link]" |
| Nudge to start next session | Participant completed session | Send a reminder to start next session | "Hi {{first name}}, Salam here! A new session is available! head to the app to go through it, [link]" |
| Inactivity 1 | Participant hasn't logged in for 5 days | Send a reminder to login and check sessions | "Hi {{first name}}, Salam here! Remember to check the app. There's plenty of content for you. [link]" |
| Inactivity 2 | Participant hasn't logged in for 10 days | Send a reminder to login and check sessions | "Hi {{first name}}, Salam here! Remember to check the app. There's plenty of content for you. [link]" |
| Reminder about the app 1 | 11 weeks from account creation | Send a reminder that the chatbot is still accessible | "Hi {{first name}}, Salam here! Did you know you can still use the website? Go to [link]" |
| Reminder about the app 2 | 15 weeks from account creation | Send a reminder that the chatbot is still accessible | "Hi {{first name}}, Salam here! This is a reminder you can still access the website if you like. Go to [link]" |
| Closing the chatbot | Notify participants about trial end | Message to all participants at the end | "Hi {{first name}}, this is Salam for the last time! I want to let you know the Salam project has ended and we will close the website on {{date}}" |
